# Assessing Residents in the Department of Surgery at a Tertiary Care Centre Using Mini-Clinical Evaluation Exercise (Mini-CEX)

**DOI:** 10.7759/cureus.58011

**Published:** 2024-04-11

**Authors:** Priyanka Rai, Apul Goel, Sanjay K Bhat, Amarjot Singh, Rohit Srivastava, Sunil Singh

**Affiliations:** 1 General Surgery, Dr. Ram Manohar Lohia Institute of Medical Sciences, Lucknow, IND; 2 Urology, King George's Medical University, Lucknow, IND; 3 Surgery, Dr. Ram Manohar Lohia Institute of Medical Sciences, Lucknow, IND

**Keywords:** mini-clinical evaluation exercise, mini-cex, assesment in medical education, medical resident education, medical education

## Abstract

Objective: This study aimed to introduce, sensitize, and train our postgraduate students and faculty of the department of general surgery with the use of mini-Clinical Evaluation Exercise (mini‑CEX) and to assess the perception of students and faculty towards it.

Material and methods: A cross‑sectional observational study was conducted over a period of four months. Ten surgery residents in the department were asked to volunteer to participate and five professors conducted the session. Five sessions of mini‑CEX (nine points) were conducted for each resident in different settings of the out‑patient department (OPD) and in‑patient department (IPD). A total of five skills were tested. Feedback from faculty and residents regarding the perception of mini‑CEX was also taken.

Results: A statistically significant difference in mean scores of all domains was observed comparing the first and last assessment (p<0.05). Hundred percent of the residents scored superior category (7-9) in the final assessment in all domains, whereas the maximum was in a satisfactory scoring grade in 1st assessment. The time taken for the assessment significantly reduced from 1st assessment to the last assessment in OPD and IPD settings (p=0.001). The mini-CEX assessment tool got 100% feedback from faculty in terms of skill improvement, method, attitude of residents, and ability to identify gaps in knowledge. However, one assessor thought that "time given for assessment" was inadequate and more effort was required than the usual traditional assessment methods. The most identified problem faced by residents was that the "time given during assessment" was less (50%); however, overall residents also found it valid, effective, and helpful in identifying knowledge gaps and improving clinical and communication skills.

Conclusion: Mini‑CEX improves the learning environment in residency and also leads to improvement in medical interviewing skills, physical examination skills, humanistic qualities/professionalism, and counseling skills. So, it can be used for residency training in clinical departments.

## Introduction

Clinical skills hold foremost importance in patient care and assessing them can be difficult and complex [1]. Presently, the majority of methods of assessments for postgraduate student (PGS) utilized in Indian medical colleges are summative in nature, focusing more on the PGS's knowledge acquisition than their clinical aptitude. These assessments include essay-style questions written yearly and summative ratings that include oral case-based discussions and table viva voce [2].

Assessment is an essential component of any education. By keeping track of students' advancement and accomplishment in relation to the curricular outcomes, it speeds up the learning process. Numerous instruments have been created to fulfill this function [3]. The mini-Clinical Evaluation Exercise is one of the most used instruments for evaluating trainees' performance in professional environments. In this, a specialist, who is usually a faculty member, watches trainees in action, evaluates the range of their clinical abilities, and gives them suitable comments [4].

Mini-Clinical Evaluation Exercise (mini-CEX), which is a hybrid tool, enables both assessment and feedback at the same time. Mini-CEX's primary advantage is its ability to deliver task-related, real-time feedback from an experienced assessor [5]. The mini-CEX, created by the American Board of Internal Medicine, can solve the majority of the assessment issues like assessing essay-type answers, one-time assessment of clinical skills, and yearly practical examination [4]. In the mini-CEX, a trainee is observed closely throughout a targeted clinical interaction, and feedback is given right away. The evaluation is documented on a rating form, the results of which are equivalent to a high-stakes clinical test and have been demonstrated to have good internal consistency and reliability among trainees in medical education [6,7]. There is more fidelity in the mini-CEX than in other formats [8].

The mini-CEX has been used extensively worldwide for a variety of demographics and situations since the American Board of Internal Medicine (ABIM) introduced it in the 1990s. Numerous articles detailing the use of the mini-CEX for formative or summative purposes were found during our scoping search. Nonetheless, these reports differ in a few areas, such as the quantity of necessary interactions, the background information provided by the raters, and the structure of the assessment form. A number of these research have focused on topics including users' satisfaction with the mini-CEX, educational effects, and psychometric features [3].

In our institution, surgery residents do not have a structured assessment program during residency, and the focus is on the summative assessment at the end of the course. So, this study was done to introduce, sensitize, and train our postgraduate students and faculty of the department of general surgery, in the use of mini-CEX. We also assessed the perceptions of post-graduate students and faculty members regarding mini-CEX as an assessment/learning tool in the department of general surgery.

## Materials and methods

A cross-sectional study was conducted over a period of four months in the general surgery department of a tertiary care center in northern India. The postgraduate program in the department of general surgery had recently begun in the study setting, so there were 10 residents present at the start of the study. So, these 10 surgery residents were asked to volunteer for participation and five professors from the same department conducted the sessions.

Data collection and tool

Five sessions of mini‑CEX were conducted for each resident in different settings of the out‑patient department (OPD) and in‑patient department (IPD), with an emphasis on not repeating the clinical cases. A verbal consent was taken from students, faculties, and patients at the time of the assessment. Both the faculty and the residents were explained in detail about the process of the new assessment being used in the study. It was taken care that each student should have at least one session in each setting, that is, OPD and IPD. The students were assessed using standard mini‑CEX proforma, testing five core clinical skills rated on a nine‑point scale. These five skills were as follows: history taking, physical examination, humanistic qualities/professionalism, clinical judgment, and counseling abilities. A score of 1-3 was considered unsatisfactory, a score of 4-6 was considered satisfactory, and a score of 7-9 was considered superior. A self-designed feedback form was used to take feedback from faculty and residents regarding the perception of mini‑CEX.

Data analysis

Data were analyzed using SPSS version 24.0 (Chicago, IL: IBM Corp.), and ANOVA (paired t-test) for inferential statistics was done. Normality of the data was tested by the Kolmogorov-Smirnov test. Descriptive statistics like mean, standard deviation, frequencies, and percentages were used to present the study results. A paired t-test was done for descriptive statistics. Categorical variables were analyzed using chi-square test. Probability will be calculated to test statistical significance at the 5% level of significance. Ethical approval was obtained from the Dr. Ram Manohar Lohia Institute of Medical Sciences (RMLIMS) Institutional Ethical Committee (IEC no. 132/23).

## Results

A statistically significant difference in mean scores of all domains, i.e., history taking, physical examination, humanistic behavior, clinical judgment, and counseling was observed comparing the first and last assessment (p<0.05) (Table 1). The score was divided into the following three categories: 1-3 unsatisfactory, 4-6 satisfactory, and 7-9 superior. Hundred percent of residents scored superior category in the final assessment in all domains, whereas the maximum scored a satisfactory scoring grade in 1st assessment (Table 2). The time taken for the assessment significantly reduced from 1st assessment to the last assessment in OPD and IPD settings (p=0.001) (Table 3).

**Table 1:** Mean scores in various domains of mini-CEX assessment at first and last exposure (p<0.05 considered significant). A statistically significant difference in mean scores of all domains, i.e., history taking, physical examination, humanistic behavior, clinical judgment, and counseling was observed comparing the first and last assessment (p<0.05).

Mini-CEX domain	1st assessment	Final assessment	p-Value
History taking	5.4±1.28	8.5±0.4	0.005
Physical education	5.6±0.97	8.5±0.5	0.0004
Humanistic/professional behavior	6.6±0.92	8.6±0.4	0.002
Clinical judgment	6.1±0.7	8.7±0.25	0.010
Counseling	5.7±0.9	8.7±0.3	0.0053

**Table 2:** Distribution of study participants on the basis of grading of scores at first and last exposure. The score was divided into the following three categories: 1-3 unsatisfactory, 4-6 satisfactory, and 7-9 superior. Hundred percent of the residents scored superior category in the final assessment in all domains, whereas the maximum was in a satisfactory scoring grade in 1st assessment.

Mini-CEX domain		Unsatisfactory	Satisfactory	Superior
History taking	1st assessment	1 (10%)	7 (70%)	2 (20%)
	Final assessment	0	0	10 (100%)
Physical education	1st assessment	0	7 (70%)	3 (30%)
	Final assessment	0	0	10 (100%)
Humanistic/professional behavior	1st assessment	0	3 (30%)	7 (70%)
	Final assessment	0	0	10 (100%)
Clinical judgment	1st assessment	0	7 (70%)	3 (30%)
	Final assessment	0	0	10 (100%)
Counseling	1st assessment	0	8 (80%)	2 (20%)
	Final assessment	0	0	10 (100%)

**Table 3:** Mean time taken for first and last exposure assessment in both study setting areas (p<0.05 considered significant). The time taken for the assessment significantly reduced from 1st assessment to the last assessment in OPD and IPD settings (p=0.001).

Variables		Mean time (seconds)	p-Value
OPD	1st assessment	340±40	0.001
	Final assessment	260±50	
IPD	1st assessment	510±50	0.001
	Final assessment	410±40	

**Figure 1:**
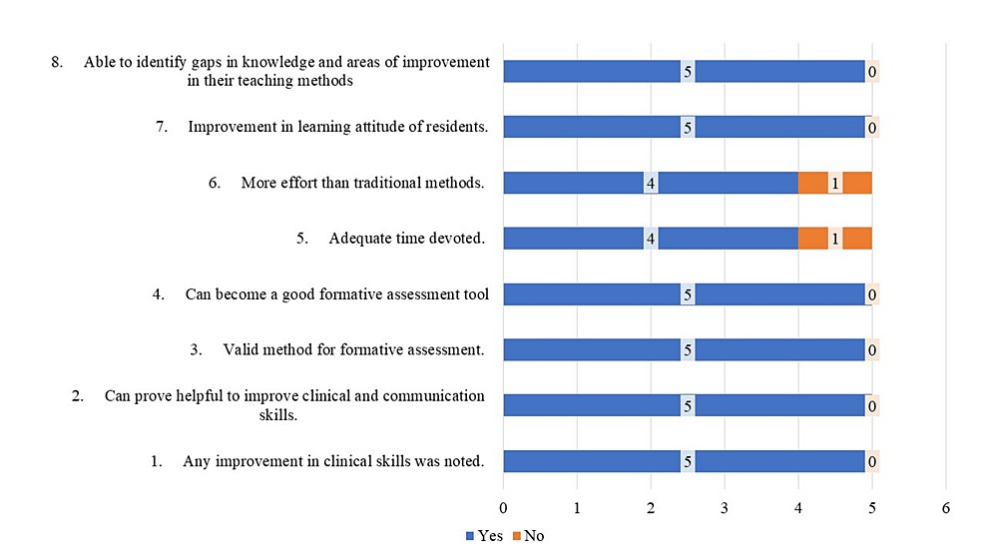
Faculty feedback on mini-CEX assessment. Feedback from faculty in terms of skill improvement, method, attitude of residents, and ability to identify gaps in knowledge. A total of 20% thought that the time given was inadequate and more efforts were required than the usual traditional assessment methods. Mini-CEX: mini-Clinical Evaluation Exercise

**Figure 2:**
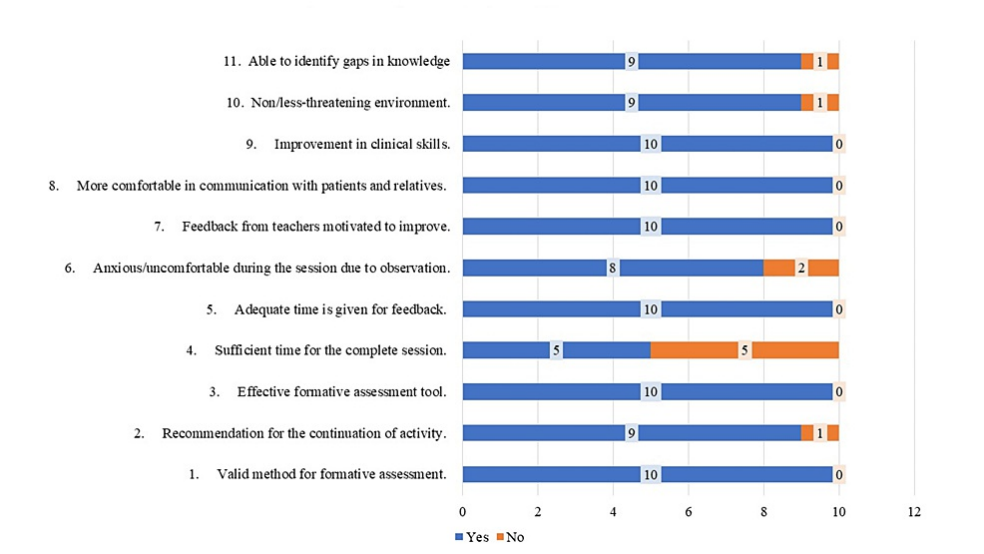
Resident feedback on mini-CEX assessment. Only time given during assessment was the most identified problem area among residents (50%); however, overall residents also found it valid, effective, and helpful in identifying knowledge gaps and improving clinical and communication skills. Mini-CEX: mini-Clinical Evaluation Exercise

Considering the feedback of residents and faculty, the mini-CEX assessment tool got 100% feedback from faculty in terms of skill improvement, method of assessment, attitude of residents, and ability to identify gaps in knowledge. However, 20% (one assessor) thought that the time given was inadequate and more efforts were required than the usual traditional assessment methods (Figure 1). The most identified problem faced by residents was that the "time given during assessment" was less (50%); however, overall residents also found it valid, effective, and helpful in identifying knowledge gaps and improving clinical and communication skills (Figure 2).

## Discussion

One of the most widely utilized workplace-based assessments (WBAs) is the mini-Clinical Evaluation Exercise (mini-CEX), which has been included in undergraduate and graduate programs all across the world since its launch in 1995 [9-13]. Trainees are watched and assessed during history taking and physical examinations, which follow structured feedback [4,14]. With the mini-CEX, trainees may get feedback from many supervisors on a variety of clinical situations and work environments [14]. As an evaluation instrument, the mini-CEX is still one of the most researched WBAs in terms of validity and reliability [9].

Usually, eight to 10 encounters are thought to be sufficient for satisfactory dependability; however, the precise number will obviously depend on the assessment's goals and stakes [15]. The close correspondence between assessment and practice setting limits validity threats such as construct-irrelevant variance and construct underrepresentation [15]. Positive associations have also been consistently found with other assessment outcomes, including high-stakes national specialty examinations [13,15-18]. Furthermore, some studies show improvements in scores over the course of the academic year or greater scores with each year of postgraduate study [10,15,19,8]. On the other hand, the mini-CEX's scoring components have drawn criticism [15]. Some of these include leniency of the rater and individual competencies affecting the rating and assessment standards of the assessor [20].

In our study, 76% of sessions were conducted in OPD and 24% were conducted in IPD. These outcomes were in good agreement with those of Norcini et al. where 38% of sessions were with ambulatory patients, 54% in IPD, and 14% in the emergency room (ER) (4% had missing information in the study setting) [4]. In another research by Singh and Sharma, 25% of sessions were held in an IPD environment and 75% of sessions were held in an ambulatory setting (OPD) [5]. Half of the sessions done by Batra et al. were ambulatory patients, whereas the remaining 30% involved inpatients and 20% in the ER [21].

In the present study, we found statistically significant improvement in mini-CEX scores of first and last assessment exposure. Some studies have been conducted to assess the use of mini-CEX assessment in various fields of medical education. Similar to our study, they all have found the effective use of mini-CEX in undergraduate [3,22-25] and postgraduate medical education [3,21,26,27].

Mini-CEX assessment in general surgery residents was also assessed by Batra et al. [21]. For medical interviewing abilities, humanistic traits/professionalism, clinical judgment, counseling, and overall clinical competency, the study's mean score was 5. For physical examination skills and organizational efficiency, the mean score was 4. According to Norcini et al., the mean score for clinical judgment, physical examination, and medical interviewing abilities was 6.5; for humanistic traits, it was 7, and for total competence, it was 6.5 [4]. The study conducted by Chang et al. revealed that the average score for medical interviewing skills was 6.6 [28]. Additionally, the mean scores for physical examination, clinical skills, counseling skills, organization/efficiency, and clinical judgment were 6.5, 6.7, and 6.8, respectively.

To evaluate the viability, dependability, and acceptability of deploying the mini-CEX in the emergency room, a comprehensive review and meta-analysis were conducted [27]. The average duration of trainee-patient encounters in the four included publications was 16.05 minutes (95% CI: 14.21-17.88), and the average time for providing feedback was 10.78 minutes (95% CI: 10.19-11.38). Our mean interaction time was quite less in the last encounter than in the first one. In the feedback from faculty and residents "time spent for the process" was the most commonly reported drawback. However, there was a statistically significant difference in mean time of both encounters indicating that once the trainer-trainee got used to the process of assessment less time was taken for the same.

Overall, professors and residents responded very well to mini-CEX. According to a research, feedback content has the potential to enhance mini-CEX's usefulness as an assessment tool and provide both constructive and critical feedback [29]. Positive feedback about the use of mini-CEX in many areas of medical education has been received by numerous other researchers [4,21,30,31].

Similarly, constructive feedback from both the assessor and the student has been shown to have an impact on a student’s education [30]. Thus, mini‑CEX supports feedback which can improve the performances of the trainee, though leniency of assessors might limit the identification of trainees who underperform [31]. This was also the problem faced in our study, where, in the initial assessments, the faculty was a little reluctant to give unbiased scores but later on after being acquainted with the pattern they evaluated impartially, and the sessions were carried out till the complete satisfaction of the assessor.

The strength of the study lies in the fact that mini‑CEX can be used as a tool for assessment as well as teaching clinical skills as a part of the workplace‑based assessment. Because the assessor provides quick feedback, it may be utilized as a teaching tool to help residents improve their varied abilities for future clinical interactions. It may be used as a clinical tool since it contains a clearly specified proforma that covers every facet of the clinical interaction. This study has certain limitations, including fewer residents, a short study period, and a failure to take into account the correlation between case complexity and score.

## Conclusions

Mini-CEX is a useful and appropriate instrument for evaluating residents, particularly for formative evaluation. It enhances the residency learning environment and promotes the development of medical interviewing, physical examination, humanistic and professional skills, and counseling abilities. Since it was done during a real patient encounter, it should help the residents deal with patients more effectively in the future. Nonetheless, a greater proportion of educators believed that mini-CEX needed more work than conventional techniques.
